# Exploring the Inflammatory Metabolomic Profile to Predict Response to TNF-α Inhibitors in Rheumatoid Arthritis

**DOI:** 10.1371/journal.pone.0163087

**Published:** 2016-09-15

**Authors:** Bart V. J. Cuppen, Junzeng Fu, Herman A. van Wietmarschen, Amy C. Harms, Slavik Koval, Anne C. A. Marijnissen, Judith J. W. Peeters, Johannes W. J. Bijlsma, Janneke Tekstra, Jacob M. van Laar, Thomas Hankemeier, Floris P. J. G. Lafeber, Jan van der Greef, on behalf of all Society for Rheumatology Research Utrecht investigators

**Affiliations:** 1 Rheumatology & Clinical Immunology, University Medical Center Utrecht, Utrecht, The Netherlands; 2 Leiden Academic Center for Drug Research, Leiden University, Leiden, The Netherlands; 3 Sino-Dutch center for Preventive and Personalized Medicine, Zeist, The Netherlands; 4 TNO, Netherlands Organization for Applied Scientific Research, Microbiology & Systems Biology, Zeist, The Netherlands; 5 Netherlands Metabolomics Center, Leiden, The Netherlands; 6 St Jansdal hospital, Harderwijk, The Netherlands; Korea University, REPUBLIC OF KOREA

## Abstract

In clinical practice, approximately one-third of patients with rheumatoid arthritis (RA) respond insufficiently to TNF-α inhibitors (TNFis). The aim of the study was to explore the use of a metabolomics to identify predictors for the outcome of TNFi therapy, and study the metabolomic fingerprint in active RA irrespective of patients’ response. In the metabolomic profiling, lipids, oxylipins, and amines were measured in serum samples of RA patients from the observational BiOCURA cohort, before start of biological treatment. Multivariable logistic regression models were established to identify predictors for good- and non-response in patients receiving TNFi (n = 124). The added value of metabolites over prediction using clinical parameters only was determined by comparing the area under receiver operating characteristic curve (AUC-ROC), sensitivity, specificity, positive- and negative predictive value and by the net reclassification index (NRI). The models were further validated by 10-fold cross validation and tested on the complete TNFi treatment cohort including moderate responders. Additionally, metabolites were identified that cross-sectionally associated with the RA disease activity score based on a 28-joint count (DAS28), erythrocyte sedimentation rate (ESR) or C-reactive protein (CRP). Out of 139 metabolites, the best-performing predictors were *sn1*-LPC(18:3-ω3/ω6), *sn1*-LPC(15:0), ethanolamine, and lysine. The model that combined the selected metabolites with clinical parameters showed a significant larger AUC-ROC than that of the model containing only clinical parameters (p = 0.01). The combined model was able to discriminate good- and non-responders with good accuracy and to reclassify non-responders with an improvement of 30% (total NRI = 0.23) and showed a prediction error of 0.27. For the complete TNFi cohort, the NRI was 0.22. In addition, 88 metabolites were associated with DAS28, ESR or CRP (p<0.05). Our study established an accurate prediction model for response to TNFi therapy, containing metabolites and clinical parameters. Associations between metabolites and disease activity may help elucidate additional pathologic mechanisms behind RA.

## Introduction

Rheumatoid arthritis (RA) is a chronic, disabling disease that mainly affects the synovial joints. The disease is a multifactorial autoimmune disorder with a prevalence of 0.5–1% in industrialized countries [1–3]. Disease-modifying anti-rheumatic drugs (DMARDs) are the cornerstone of anti-inflammatory therapy in RA and can be divided into two categories: conventional synthetic DMARDs (csDMARDs) and biological DMARDs (bDMARDs) [4]. The csDMARDs are relatively cheap chemical agents consisting of small active-substance molecules and are used for decades in the treatment of RA, whereas bDMARDs, predominantly antibodies such as TNF-α inhibitors (TNFis), are expensive agents that target specific inflammatory pathways and have revolutionized the treatment options for RA patients since the first trial in 1993 [5]. Despite the success of TNFis, a substantial proportion of patients (approximately 30–40%) responds insufficiently to these biological agents [6,7]. At the initiation of TNFi therapy it is as yet impossible to distinguish future responders from non-responders, therefore, the only used treatment approach is by trial and error. This approach is inefficient because the clinical response can only be assessed after at least three months of treatment. Within this timeframe, non-responders might develop joint damage or may experience toxic side effects. In addition, an inefficient treatment increases healthcare costs due to intensive monitoring, more complications, higher morbidity, and medication costs. The challenge is therefore to identify responders and non-responders before initiation of TNFi treatment so that the decision can be guided and the most optimal agent can be selected for each patient. Many approaches have been explored, mostly by evaluation of clinical parameters, proteins or mRNA biomarker profiles, but none were thus far successful in such a way that they can be implemented in clinical practice [8].

Metabolomics is a rapidly developing approach in biomarker research, involving the measurement of a large number of small-molecule metabolites in biological fluids, tissues and cells. One major advantage is that it offers a characteristic profile of each patient from minimal amounts of sample. With high-throughput techniques, such as nuclear magnetic resonance (NMR) spectroscopy and liquid chromatography coupled to mass spectrometry (LC-MS), metabolite profiles in disease or therapeutic response to treatment can be measured [9]. In this sense, metabolomics provides a novel perspective on the search of new disease biomarkers and drug targets.

Metabolomic approaches in RA have already contributed to the understanding of RA and its subtypes, as well as the effect of drug treatment [10]. However, there is a limited number of studies employing metabolomic profiling to predict patients’ response to biological therapies. To our knowledge, only two previous studies have used metabolomics to predict the clinical response to TNFi, both by usage of the ^1^H-NMR-based technique. Kapoor et al. [11] screened the urine metabolome of 16 RA patients and found that histamine, glutamine, xanthurenic acid, and ethanolamine predicted TNFi response. In addition, there was a significant correlation between baseline urine metabolic profile and the magnitude of the one-year change in the disease activity. Priori et al. [12] showed that the serum metabolic profiling of 27 RA patients at baseline could discriminate the response to etanercept. Additionally, higher levels of isoleucine, leucine, valine, alanine, glutamine, tyrosine, and glucose and lower levels of 3-hydroxybutyrate were observed in good responders after 6 months of therapy. The predictors found in these ^1^H-NMR based studies have thus far not been validated in other cohorts.

In the present study, the baseline serum metabolome of RA patients commencing biological therapy was analyzed using LC-MS, a different technique as previous studies used, targeting a large scope of metabolites consisting of lipids, oxylipins, and amines. The objective was to assess the potential value of serum metabolite profiles in the prediction of response to TNFi treatment, using LC-MS. Additionally, we investigated the association between metabolites with current disease activity, in order to gain more insight into the pathologic metabolic mechanisms in RA.

## Materials and Methods

### Patient cohort

Patients were selected from the observational BiOCURA study (Biologicals and Outcome, Compared and predicted in Utrecht region, in Rheumatoid Arthritis) in which patients were enrolled between 2009 and 2015. In BiOCURA, RA patients eligible for bDMARD treatment in clinical practice, were followed up after start of treatment, in one academic hospital and seven regional hospitals in the Netherlands (see Acknowledgments). The treatment included any of five TNFis, adalimumab, etanercept, infliximab, golimumab, and certolizumab pegol, or non-TNFi agents, including tocilizumab, abatacept, and rituximab. All csDMARDs were allowed to be used concomitantly with the bDMARD, and included methotrexate (MTX), hydroxychloroquine (HCQ), leflunomide (LEF), and glucocorticoids (GCs). Apart from csDMARDs, patients continued other medication, such as statins, bisphosphonates, anti-hypertension and nonsteroidal anti-inflammatory drugs (NSAIDs), all according to regular clinical practice.

Regular visits with the treating physician and a trained research nurse were scheduled at baseline, three, six, and twelve months. Clinical parameters and blood samples were collected by the nurse from each patient before the first dose of the biological agent. Of note, blood samples were collected from fasting and non-fasting patients, as the visiting times of the patients could not be standardized in the morning. The blood was collected in a Vacutainer® SST II tube and processed immediately after clotting. Samples were centrifuged for 10 min at 1500 *g* at room temperature and serum was aliquoted and stored at -80°C until use for metabolomic analyses. Re-inclusion after switching to a different biological agent was possible. The study was approved by the ethics committee of the UMC Utrecht and the institutional review boards of the participating centers (see Acknowledgments). Written informed consent was obtained from each patient.

Inclusion in the present study was restricted to subjects of BiOCURA fulfilling the following criteria: at start of treatment patients should not be in clinical remission (disease activity score based on a 28-joint count, DAS28 > 2.6), after three months of therapy the DAS28 assessment needed to be available, and no (temporary) discontinuation of treatment should have occurred within the first three months of bDMARD treatment.

### Clinical measurements

Demographic, clinical, and laboratory parameters of patients at baseline were obtained, including age, gender, menopausal status, body mass index (BMI), disease duration, any previously used bDMARD (biological naivety), currently used csDMARDs and non-anti-rheumatic drugs, 28 tender joint count (TJC), 28 swollen joint count (SJC), a 100mm visual analogue scale on general health (VAS-GH), erythrocyte sedimentation rate (ESR), C-reactive protein (CRP), rheumatoid factor (RF), and anti-citrullinated protein antibody (ACPA). Disease activity was assessed at baseline and at follow-up visits, using DAS28 [13]. In clinical practice the response to biological therapy is usually measured 3–6 months after initiation [14]. However, in BiOCURA a substantial number of patients withdrew treatment before the 6-month time-point due to insufficient response or side effects. Using the 6-month response would thus result in (non-random) missing responses. Therefore, in this study, the patients’ response was determined after 3-month of treatment, based on the EULAR response criteria [15]. A EULAR good response is defined as an improvement in DAS28 of > 1.2 and a present DAS28 ≤ 3.2, whereas a EULAR non-response is assigned to patients with an improvement of 0.6–1.2 with present DAS28 > 5.1 or patients with an improvement ≤0.6. In between, an improvement > 1.2 with present DAS28 > 3.2 or an improvement of 0.6–1.2 with present DAS28 < 5.1 is specified as a EULAR moderate response.

### Metabolomic profiling

Serum samples from selected subjects were measured on three targeted LC-MS platforms, which used standard operating procedures from previously published methods [16–18], covering a broad spectrum of pre-defined metabolites. The lipid platform targets low abundance lipid species, including free fatty acids (FAs) and phospholipid derivates, such as lysophosphatidylcholines (LPCs) and lysophosphatidylethanolamines (LPEs); the oxylipins platform covers oxygenated metabolites derived from polyunsaturated fatty acids through enzymatic and non-enzymatic oxidation processes; the amine platform targets amino acids and biogenic amines. All analyses were performed by the Biomedical Metabolomics Facility Leiden (BMFL) of the Leiden University. Extra serum of the subjects was pooled and used to create internal quality control (QC) samples.

#### Lipids analysis

For the detection of lipids, each 20 μL serum aliquot was spiked with internal standard (ISTD) mix and lipids were extracted by methanol. This lipid profiling was conducted using ultra performance liquid chromatography coupled to electrospray ionization-quadrupole time-of-flight (Agilent 6530 San Jose, CA, USA) with an ACQUITY UPLC™ HSS T3 column (1.8 μm, 2.1×100mm) [16].

#### Oxylipins analysis

Each 180 μL serum aliquot was spiked with antioxidants and ISTD mix, followed by solid phase extraction. The samples were analyzed by high-performance liquid chromatography (Agilent 1260, San Jose, CA, USA) coupled to a triple quadrupole mass spectrometer (Agilent 6460, San Jose, CA, USA), using an Ascentis® Express column (2.7 μm, 2.1x150 mm) [17].

#### Amines analysis

Each 5 μL serum aliquot was spiked with an ISTD mix and proteins were precipitated by methanol, after which the supernatant was dried and derivatized by AQC reagent. The samples were analyzed by an ACQUITY ultra-performance liquid chromatography system coupled to Xevo Tandem quadrupole mass spectrometer (Waters, Milford, MA, USA) with an AccQ-TagTM Ultra column (1.7 μm, 2.1x100 mm) [18].

#### Data preprocessing and correction

For the lipid and oxylipin platforms, peak determination and peak area integration were performed by Mass Hunter Quantitative Analysis (version B.05.00, Agilent technologies); for the amine platform, TargetLynx software (version 4.1, Waters) was employed. For each metabolite, the concentration was determined by the ratio between the peak area of targeted analyte and peak area of the appropriate ISTD. These response ratios (Area _analyte_/Area _ISTD_) were used as raw metabolomic data in the subsequent analysis. For batches measured in each platform, an in-house developed method was applied to compensate and correct for instrumental drift during the measurements. The within-batch and between-batch effects of metabolomic data were corrected per metabolite using the responses of QC samples [19]. The QC samples were measured repeatedly every 10 patient samples across the different batches per platform. The details of the data correction method are described by van der Kloet *et al* [20]. The relative standard deviation (RSD) of metabolites in the QC samples was used to assess the quality of targeted metabolites in each analytical platform.

Metabolite measurements, which were lower than the limitation of detection (LOD) of the platform, were imputed by half of the observed minimum value for the corresponding metabolite. Subsequently, log-transformation and auto-scaling of the metabolites were applied to reach or approximate a normal distribution, with a mean of 0 and SD of 1. The resulting data were used as input for all subsequent statistical analyses.

### Statistical analyses

**Fig 1:**
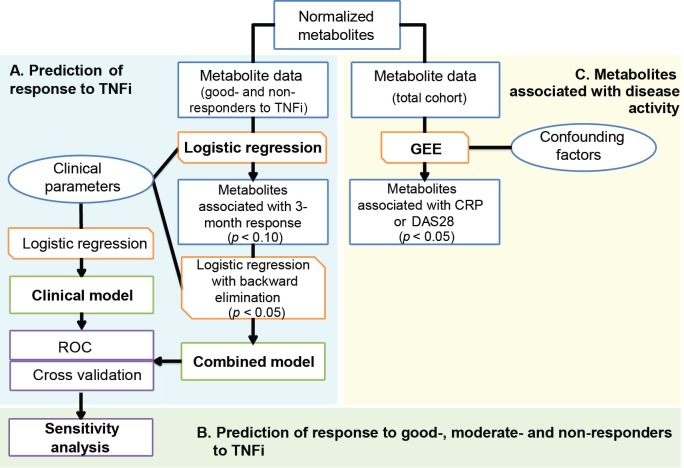
Flowchart of statistical analyses. (A) Prediction of response to TNFi: All steps to build a prediction model on TNFi response were performed on the TNFi subset with EULAR good-response or non-response (n = 124). (B) Sensitivity analysis on the complete cohort of TNFi initiating patients. (C) Metabolites associated with disease activity. Analyses to investigate metabolites association with CRP, ESR or DAS28 were performed on the total cohort of patients using bDMARDs (n = 231; including TNFi and non-TNFi treated patients). Blue boxes/circles indicate (selection of) respectively metabolites or clinical parameters, whereas orange boxes indicate the performed analyses. bDMARDs: biological disease-modifying anti-rheumatic drugs; CRP: C-reactive protein; DAS28: disease activity score based on a 28-joint count; ESR: erythrocyte sedimentation rate; GEE: generalized estimating equation, LC-MS: liquid chromatography coupled to mass spectrometry; ROC: receiver operating characteristic; TNFi: TNF-α inhibitor.

Metabolites were used to develop a model for the prediction of response to TNFi at 3 months, and to assess their association with disease activity in general of the total cohort. An overview of the analyses is provided in Fig 1 and will be discussed in more detail below. All analyses were performed in IBM SPSS Statistics for Windows, Version 22.0 (IBM Corp., Armonk, N.Y., USA), MedCalc for Windows, version 16.2.1 (MedCalc Software, Ostend, Belgium),and R (Version 3.2.3).

#### Development of models for predicting TNFi response

In order to increase the possibility of picking up predictors related to response, we focused on subjects with EULAR good- *versus* non-response to TNFi treatment only, since EULAR moderate responders are an in-between category in which some patients continue treatment and others discontinue due to inefficacy (BiOCURA data). With TNFi good responders and non-responders as an outcome, we built two multivariable logistic regression models: a clinical model including clinical parameters only and one combined model with the same clinical parameters and the metabolites. We selected the following baseline clinical parameters: age, gender, menopausal status, BMI, smoking status, alcohol consumption, DAS28, (log-transformed) CRP, concomitant csDMARDs (MTX, HCQ, LEF, GCs) and non-DMARDs (statins, antihypertensive drugs, bisphosphonates and NSAIDs), regardless of the predictive ability (i.e. p-value) of each single parameter.

To decrease the number of metabolites in the combined model, a (non-strict) pre-selection on metabolites was performed. The predictive value of each individual metabolite to TNFi treatment outcome (good- or non-response) was investigated while combined with the clinical parameters, and all metabolites with *p* < 0.10 were pre-selected. Subsequently, the pre-selected metabolites together with the clinical parameters were added to the logistic regression model on response with backward elimination of the metabolites, to build the final combined model (thus keeping all clinical parameters in).

The receiver operating characteristic (ROC) curves of the clinical and combined model was plotted, with the area under the curve (AUC) as an indicator of its predictive ability. Sensitivities, specificities, misclassification rates (MR), and positive- and negative predictive values (PPV/NPV) were calculated based on the optimal cutoff (Youden’s index [21]). This optimal cut-off was applied to both models, so that per patient per model, either a non-response or good response was predicted, which could be compared to the observed response. Based on these predicted and observed responses, the net reclassification index (NRI) was calculated to determine if the addition of metabolites reclassifies more patients into the correct category. For example, when a future non-responder is classified at baseline to have a high probability of good response by the clinical model, but is classified into the low probability category in the combined model, the reclassification is “more correct” and the NRI for non-responders will increase, as well as the total NRI (= NRI responders + NRI non-responders). The robustness of the final models was judged by 10-fold cross validation of the model, using the cv.glm function of the R-package boot.

As an additional sensitivity analysis, the developed models were also applied to the complete cohort of TNFi initiating patients, thus without excluding any responders. In this step, the regression coefficients of the developed clinical and combined model were frozen and used to create a prediction rule. Subsequently, this clinical and combined prediction rule were compared for their abilities to distinguish EULAR non-responders from EULAR moderate- and good responders, using the same outcome measures for predictive ability as described before.

#### Metabolites and disease activity

In order to investigate associations between metabolites and disease activity (CRP, ESR and DAS28), we analyzed the complete cohort of 231 patients with TNFi and non-TNFi therapy. However, in BiOCURA some RA patients were re-included after switching to a different biological agent (usually non-TNFi after a TNFi) and, therefore, had multiple baseline visits and follow-up periods. In order to account for the effects of subjects with multiple inclusions, generalized estimating equation (GEE) was used for these analyses, as GEE is a regression-based method that allows analyses of repeated measurements within subjects [22]. Because we were interested in the association of each individual metabolite with CRP, ESR and DAS28, and clinical characteristics of patients might influence the metabolite levels (confounding), we corrected for baseline clinical parameters except CRP, ESR and DAS28, to gain more reproducible outcomes. As such, with the (log-transformed) CRP, (log-transformed) ESR or DAS28 as the dependent variable, each individual metabolite was added into GEE as an independent variable while corrected for the possible influential factors. Cytoscape was used to visualize the significant associations [23].

## Results

In total, 231 RA patients from BiOCURA cohort fulfilled the selection criteria for the present study. Baseline characteristics of all patients are shown in S1 Table. Of all patients, 173 (74.9%) received TNFi treatment and 58 patients (25.1%) received a non-TNFi treatment. Concomitant csDMARDs and non-DMARDs were very diverse, and frequently included MTX (166 patients; 72%), GCs (93 patients; 40%) and bisphosphonates (101 patients; 44%) (S2 Table). Only 14 patients (6.1%) used no concomitant csDMARDs. The baseline characteristics of patients receiving TNFi and comparisons between non- and good responders are shown in Table 1. Among these patients, 64were EULAR non-responders and 60 were good-responders. The number of RF positive patients and the SJC was significantly higher in the good responders, besides there were no significant differences in other baseline clinical variables between good responders and non-responders. Missing data was present for < 5% for each of the variables.

**Table 1:** Baseline characteristics of all selected TNFi initiating subjects (n = 173), and split for all EULAR non-responders (n = 64) and good responders (n = 60).

	Subjects with TNFi (n = 173)	Non-responders (n = 64)	Good responders (n = 60).	*p*-value
**Female, n (%)**	130 (75.1)	50 (78.1)	43 (71.1)	0.41
**Menopausal status of females, n (%)**				0.84
Pre-menopause	40 (30.8)	16 (32.0)	16 (37.2)	
Post-menopause	82 (63.1)	28 (56.0)	26 (60.5)	
Unknown	8 (6.1)	6 (12.0)	1 (2.3)	
**Age, years, mean (SD)**	54.6 (12.4)	53.7 (13.2)	53.9 (11.7)	0.95
**Disease duration, years, median (IQR)**	6.0 (2.0–12.0)	5.0 (2.0–11.5)	5.5 (2.0–11.0)	0.95
**Smoking, currently, n (%)**	42 (24.3)	17 (26.6)	15 (25.0)	0.84
**Alcohol, >7 units/week, n (%)**	31 (17.9)	8 (12.5)	12 (20.0)	0.24
**BMI, kg/m**^**2**^**, mean (SD)**	26.8 (5.0)	27.3 (5.2)	26.5 (5.2)	0.42
**Positive RF, n(%)** ^**a**^	114 (65.9)	39 (60.9)	47 (78.3)	0.04
**Positive ACPA, n(%)** ^**a**^	123 (71.1)	41 (64.1)	46 (76.7)	0.13
**CRP, mg/dL, median (IQR)**	6.0 (3.0–13.0)	5.0 (2.8–10.0)	8.0 (3.8–15.0)	0.12
**Baseline DAS28, mean (SD)**	4.5 (1.1)	4.3 (1.2)	4.6 (0.9)	0.14
TJC, median (IQR)	7.0 (2.0–13.0)	6.5 (1.3–13.8)	6.5 (3.0–11.0)	0.73
SJC, median (IQR)	2.0 (0.0–4.0)	1.0 (0.0–3.0)	2.0 (1.0–4.0)	0.02
ESR, mm/h, median (IQR)	20.5 (11.0–34.8)	18.5 (5.3–34.0)	18.0 (10.3–33.0)	0.65
VAS-GH, mean (SD)	56.5 (23.1)	56.3 (23.4)	56.0 (23.2)	0.94

^a^ The positivity for RF and ACPA was determined by the hospitals using different measurement methods and cut-offs, according to their own laboratory standards. Therefore we were not able to show an exact titer.

Descriptive statistics are expressed as number (%) for dichotomized variables, and mean ± standard deviation (SD) and median and interquartile range (IQR) for respectively normally and non-normally distributed variables. The p-value is calculated for the difference between responders and non-responders. ACPA, anti-citrullinated protein antibody; BMI, body mass index; DAS28, disease activity score based on 28 joint count; ESR, erythrocyte sedimentation rate; IQR: interquartile range; RF: rheumatoid factor; SJC, 28 swollen joint count; TJC, 28 tender joint count; VAS-GH, 100mm visual analogue scale on general health.

The metabolites were measured using three validated platforms: lipids, oxylipins, and amines, thereby giving a broad view on the serum metabolites. 25 samples (of which 10 good-, 5 moderate-, and 10 non-responders) could not be analyzed in oxylipins platform due to inadequate serum volume. After analysis and data pre-processing, one sample in lipid platform and one sample in amine platform were excluded due to abnormal ISTD areas. For each platform, RSDs of QC samples were applied as quality indicators. In total, 139 metabolites were measured: 40 amines with QC RSD <0.15, 68 lipids (lysophospholipids and fatty acids) with QC RSD < 0.30, and 31 oxylipins both with QC RSD < 0.30 (S3 Table). The details of targeted compounds are shown in S4–S6 Tables.

### Metabolites and TNFi response

In the TNFi cohort, the clinical and combined model were built for the prediction of response containing all selected 16 clinical baseline parameters (as listed in the Materials and Methods section “Development of models for predicting TNFi response”). We did not exclude any clinical parameters, for three reasons. First, because the comparison of the clinical and combined model containing the same (complete) set of clinical parameters, allows us to compare the sole added value of metabolites over clinical parameters alone. Second, when biomarkers are in fact a (partial) reflection of any clinical parameter, we avoid the incorporation of any metabolite that provides knowledge which could have been obtained with a simple clinical parameter. Third, because clinical parameters might serve as partially influential factors (i.e. confounders) on metabolism as well, incorporation of all clinical parameter in the model would result in more reliable estimates of the true predictive ability of the metabolites and thus increases the reproducibility and externalization of results.

**Fig 2:**
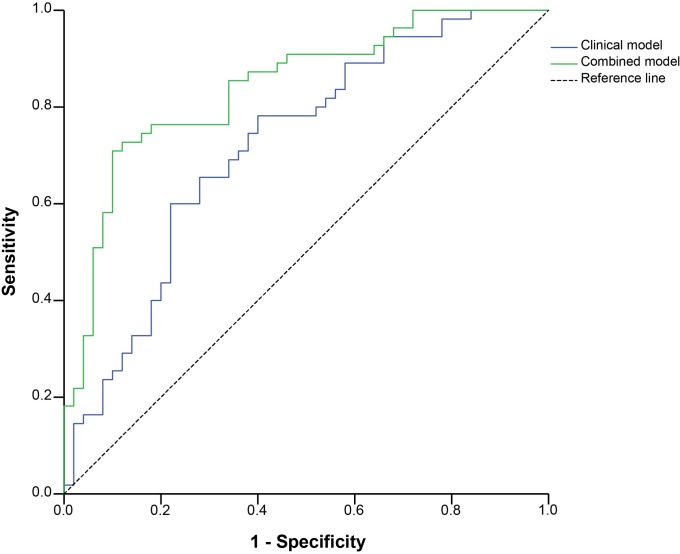
Receiver operating characteristic curves of clinical and combined model between good- and non-responder to TNFi. The clinical model containing the 16 selected baseline clinical parameters; the combined model included four metabolites with *p* < 0.05 in multivariable logistic regression with backward selection in addition to the clinical model.

The clinical model containing all selected 16 clinical baseline parameters (Cox & Snell R-square = 0.147, see S1 Fig) showed reasonable predictive abilities to differentiate good responders and non-responders, with an AUC-ROC of 0.720 (0.622–0.818) (Fig 2) and a sensitivity of 78.2%, specificity of 60.0%, PPV of 68.3%, NPV of 71.4% and MR of 30.5% (S7 Table).

Next, each metabolite was tested for their association with good and non-response to TNFi. *sn1*-LPC (18:3-ω3/ω6), *sn1*-LPC (16:1), *sn1*-LPC (15:0), *sn2*-LPC (18:1), LPE (20:3-ω3/ω6), LPE (18:1), *sn2*-LPC (18:0), LPE (18:0), *sn1*-LPC (18:0), 9,10-DiHOME, 9-HODE, 11-HDoHE, 8-HETE, 9,10-EpOME, ethanolamine, and lysine were preselected based on *p* < 0.10. Multivariable logistic regression with backward selection was carried out on preselected metabolites to establish the final combined model. Four metabolites significantly added to the prediction of clinical response to TNFi therapy (Table 2).

**Table 2:** Remaining metabolites and their estimated contribution in the prediction of response to TNFi in the final prediction model.

	Coefficient	Standard error	*p*-value	aOR (95%-CI)
***sn1*-LPC (18:3-ω3/ω6)**	-1.54	0.53	0.004	0.21 (0.08–0.61)
***sn1*-LPC (15:0)**	1.67	0.56	0.003	5.32 (1.76–16.07)
**Ethanolamine**	-1.61	0.53	0.002	0.20 (0.07–0.57)
**Lysine**	1.02	0.40	0.010	2.78 (1.27–6.09)

aOR: adjusted odds ratio; CI: confidence interval.

At baseline, good responders showed higher levels of *sn1*-LPC (15:0) and lysine, as well as lower levels of *sn1*-LPC (18:3-ω3/ω6) and ethanolamine. The combined model (Cox & Snell R-square = 0.433, see S2 Fig) showed a good overall discriminative ability with an AUC-ROC of 0.841 (0.765–0.917) (Fig 2), and its performance is significantly better than the clinical model (difference between areas = 0.121,*p* = 0.01). After determining the optimal cut-off, sensitivity was 70.9%, specificity 90.0%, PPV 88.6%, NPV 73.8% and MR 20.0% (S7 Table). The high specificity and PPV suggest that the patients classified as good responders are frequently correctly classified (low false positive rate).

Additionally an NRI was calculated in order to investigate whether the higher accuracy also results in a better reclassification of individual patients. The reclassification of non-responders by the combined model was 30% better and of responders 7% worse, resulting in a total NRI of 0.23 (Table 3). The clinical and combined models showed prediction errors of 0.303 and 0.269 respectively, which can be considered moderately robust. In the sensitivity analysis on all responders to TNFi, the clinical model performed weakly with an AUC-ROC of 0.641 (0.548–0.734), as compared to the combined model with 0.760 (0.682–0.837) (S3 Fig). The reclassification of non-responders by the combined model was 30% better and for responders 8% worse (total NRI = 0.22, S8 Table).

**Table 3:** Net reclassification index of prediction models for good- and non-responders to TNFi.

Observed response (n = 105)^a^	Predicted by clinical model	Predicted by combined model	
		Non-response	Good response
**Non-responders (n = 50)**	**Non-response**	30 (equal)	0 (worsening)
	**Good response**	15 (improvement)	5 (equal)
**Good responders (n = 55)**	**Non-response**	6 (equal)	6 (improvement)
	**Good response**	10 (worsening)	33 (equal)

An optimal cut-off for the clinical and combined model was chosen based on the Youden’s index, after which the predicted response of each patient per model was compared to the observed response. Shown are the number of patients, split for future non-responders and good responders (observed response), that were allocated at baseline to a predicted category (non-response/good-response) by both the clinical and combined model. These allocations could be correct or wrong, based on the observed response. There are four possibilities of allocations that represent an equally good or bad performance of both models (e.g.”30” represents 30 non-responders that were correctly classified as non-responders by both the clinical and combined model). Two categories denote an improvement in the prediction by the combined model: either a future non-responder that switches from response in the clinical model to non-response in the combined model (n = 15), or a future responder switching from non-response to response (n = 6). The two remaining discordant categories denote a worsening of prediction by the combined model. The NRI for non-responders was 15/50–0/50 = 30% improvement, while the NRI for responders was 6/55–10/55 = -7% due to a net worsening in prediction by the combined model. The total NRI was 0.30 + (-0.07) = 0.23.

^a^ Due to the missing data of the clinical parameters, 19 out of 124 patients initiating TNFi therapy were excluded from the multivariable logistic regression models (clinical and combined model). Thus, 105 patients remained in the analyses.

### Metabolites and disease activity

**Fig 3:**
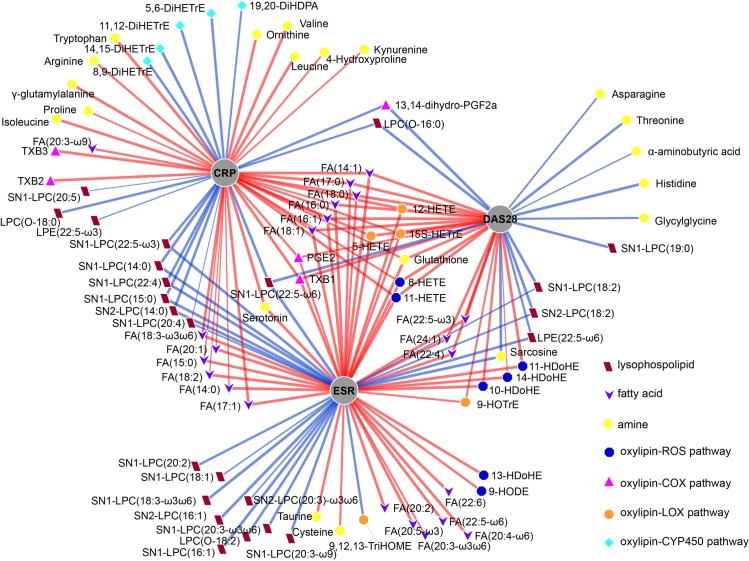
Visualization of the associations between metabolites and disease activity − general inflammation (log-transformed CRP and ESR) and RA-specific inflammation (DAS28)–based on the complete cohort of bDMARD users (n = 231). The metabolites that associated with either CRP, ESR or DAS based on linear generalized estimating equations (GEE), were grouped according to metabolic classes (LPCs, FAs, amines and oxylipins), which are represented as color-coded symbols adjacent to the metabolites. The metabolites in these metabolic classes showed comparable associations with CRP, ESR and/or DAS28. FAs positively- and the lysophospholipids negatively associated with CRP, ESR and/or DAS28; the association between other the oxylipins and amines with CRP, ESR and/or DAS28 were mixed, based on their metabolic functions. Positive associations are indicated with red lines, negative associations with blue lines; thicker lines indicate a more significant association. CRP, C-reactive protein; DAS28, disease activity score based on 28 joint counts; ESR: erythrocyte sedimentation rate; FA, fatty acid; LPE, lysophosphatidylethanolamine; LPC, lysophosphatidylcholine

Metabolites were investigated for their association with disease activity. In total, 88 metabolites out of 139 were significantly associated with baseline DAS28, ESR or CRP (*p* < 0.05) (S9 Table). In Fig 3, the associations between metabolites and the three clinical parameters are visualized. With respect to the amines, glutathione was positively associated with all the three parameters; sarcosine was negatively associated with both DAS28 and ESR, whereas serotonin was positively associated with CRP and ESR. Besides from the overlapping amines, five amines were negatively associated with baseline DAS28 (histidine, threonine, glycylglycine, asparagine, and α-aminobutyric acid), while ten amines showed a positive association with CRP (valine, leucine, tryptophan, γ-glutamylalanine, isoleucine, ornithine, arginine, 4-hydroxyproline, kynurenine, and proline) and two amines a positive association with ESR (taurine, cysteine). Fatty acids with aliphatic tails of 14 to 24 carbons were cross-sectionally found positively associated with CRP, ESR and/or DAS28, while lysophosphatidylethanolamines (LPEs) and lysophosphatidylcholines (LPCs) overall showed negative correlations with these parameters. Oxylipins were divided into different classes by the pathways—the auto-oxidation pathway with reactive oxygen species (ROS) and the enzymatic pathways with cyclooxygenase (COX), lipoxygenase (LOX) and cytochrome P450 (CYP450). Specifically oxylipins synthesized by ROS (10-HDoHE, 11-HDoHE, 11-HETE, 14-HDoHE, 8-HETE, 13-HDoHE, 9-HODE), COX (PGE2, TXB1, TXB2, TXB3) and LOX (12-HETE, 15S-HETrE, 5-HETE, 9-HOTrE) were cross-sectionally positively associated with CRP, ESR or DAS28, whereas only two downstream products in COX and LOX pathways, 13,14-dihydro-PGF2a and 9,12,13-TriHOME respectively, were found to be negatively associated with the clinical parameters.CYP450 synthesized oxylipins (11,12-DiHETrE, 14,15-DiHETrE, 19,20-DiHDPA, 5,6-DiHETrE, 8,9-DiHETrE) were all negatively associated with CRP.

## Discussion

One aim of the study was to explore the possibility of using baseline metabolomic profiling in the prediction of good- and non-response to TNFi treatment in patients with RA. Our combined model was able to predict response after 3 months of treatment with high accuracy (AUC-ROC 0.841) and with a moderate robustness (prediction error: 0.269), and was significantly better than a model containing clinical parameters alone (increase in AUC-ROC = 0.121, *p* = 0.01, NRI = 0.23). The metabolites contributing to baseline prediction were *sn1*-LPC (15:0), *sn1*-LPC (18:3-ω3/ω6), ethanolamine, and lysine, and the high NRI for non-responders indicates that these metabolites are especially useful to identify non-responders in advance. Additionally, our sensitivity analysis confirmed the added effect of metabolites in the prediction of (non-)response in all patients initiating TNFi. When the prediction rule is further validated, non-responders can be identified and offered more suitable treatments, which prevents joint damage, potentially toxic side-effects and saves healthcare costs.

Two of the identified predictors were also found to predict response in the two previous studies [11,12]. A lower level of ethanolamine was also associated with response to TNFi in the study by Kapoor et al.[11], however, opposite results for a higher level of lysine were found, which predicted good response in our study and non-response in the study by Priori et al. [12]. In addition, both of these previous studies reported high values of glutamine as predictors of response to TNFi, though glutamine did not predict response in our study with- or without correction for multiple clinical parameters (*p* = 0.312 and *p* = 0.555 respectively). Several differences in study design compared to our study might explain these apparent discrepancies, such as different analytical platforms (technique and targeted panels) and statistical method applied, on top of a smaller sample size as well as the use of urine instead of serum in one of the two studies [12]. Therefore, *sn1*-LPC (15:0), *sn1*-LPC (18:3-ω3/ω6), ethanolamine and lysine hold potential in predicting response to TNFi therapy when measured with LC-MS and on top of prediction by clinical parameters.

Two amino acids, ethanolamine and lysine, predicted good response to TNFi in our study. Ethanolamine is a primary amino acid and down-regulated levels were found to be predictive for good response to TNFi in our study. It has been reported that abundance of ethanolamine was lower in synovial fluid of RA patients than in non-RA patients [24]. The derivatives of ethanolamine play important roles in many pathways. For example, cytidine diphosphate -ethanolamine can be used as a substrate for *de novo* synthesizing phospholipids phosphatidylethanolamines (PE), which are structural components of biological membranes [25]. Up-regulation of lysine was found to be predictive for good response to TNFi in the present study. Lysine is known to be related to RA treatment. A previous study that measured metabolites in 20 patients before and after three months of TNFi treatment, reported that the concentration of lysine was elevated after three months of TNFi therapy [26]. What its exact role in inflammation is remained speculative, however in animal studies, in which acute inflammatory processes were induced, it was indicated that amino acids are redirected from muscle to the liver for acute-phase protein synthesis and gluconeogenesis[27], [28]. Although absolute levels of ethanolamine and lysine were not directly found associated with DAS28 or CRP, it is possible that the relative abundance of these metabolites is to some extent informative for the inflammatory status in RA.

Interestingly, a down-regulation of *sn1*-LPC (18:3-ω3/ω6) was found in good responders, while a down-regulated *sn1*-LPC (15:0) was found in non-responders. The most common structure of LPCs is with an even-chain fatty acid on *sn1* or *sn2* position, such as *sn1*-LPC (18:3-ω3/ω6) with an 18-carbon fatty acid. However, *sn1*-LPC (15:0) carries an odd-saturated fatty acid (OCS-FA) chain. As part of lipid metabolism, LPCs and FAs can interconvert [29]. It is reported that the serum levels of OCS-FAs are associated with the consumption of dairy products and the reduced disease risks of coronary heart disease and type 2 diabetes [30], but no study has specified the function of OSC-FA chain LPCs in the human body, and the associations between LPCs and RA have not been explored. Although in the prediction models we adjusted the metabolites for multiple clinical parameters, we were unable to correct for the dietary influences or fasted/non-fasted status. We assumed that dietary variability would be equally distributed across response groups, however, the observed levels of OCS-FAs as predictors of response might indicate that this is not the case for dairy products.

Secondary aim of the present study was to explore the relation between metabolite profiles and RA disease activity in general while correcting for most important clinical parameters. We found 88 individual metabolites related to CRP, ESR or DAS28, among which LPCs and FAs were abundant. We found that LPCs were negatively associated with all three parameters, which is a consistent finding across several studies in humans [31,32]. LPCs are lysophospholipids that play important roles in pathological processes as signaling molecules [33,34]. The *sn1*-LPC is generated by the hydrolysis of phosphatidylcholine (PC) by phospholipase A2 (PLA2). These LPCs have been studied extensively and their pro- or anti-inflammatory role and magnitude of effect are dependent on the length and (un)saturation of the fatty acyl group [35–37]. As the precursor of LPC, PC is present in the cell membrane and can bind to CRP to initiate host defense [38]. Long chain FAs are precursors of pro- and anti-inflammatory molecules [17], [39], which were found positively associated with CRP, ESR and DAS28 in this study. These results indicate that the FA metabolism was more active in the RA patients with higher disease activity and inflammation. Oxylipins generated by COX, LOX, and ROS pathway have potent pro-inflammatory effects and were positively associated with CRP or DAS28 while oxylipins derived from the CYP450 pathway were negatively associated with CRP, which can be explained by their anti-inflammatory effects [40].

In our study, several amines were found to be significantly associated with DAS28 (6 out of 7 negatively), CRP (all 12 positively) and ESR (4 out of 5 positively). Of those amines negatively related with DAS28, histidine, asparagine, and threonine were reported to be downregulated in RA patients compared to healthy controls [26], [10]. If these markers are indeed RA-specific, it is not surprising they also signify disease severity to a certain extent, which is indeed what can be concluded from the (negative) relation to DAS28 observed in our study. Amino acids in arginine metabolism (arginine and ornithine), tryptophan metabolism (serotonin and tryptophan) and branched-chain amino acids (isoleucine, leucine, and valine) were found to be positively associated with CRP, which may indicate as was suggested previously, that muscle proteins are degraded to amino acids and are redirected to the liver for acute-phase protein synthesis and gluconeogenesis [27,28,41,42]. Serotonin and taurine, which were positively associated with CRP or ESR, were reported associated with oxidative stress and could therefore be linked to the inflammatory processes in RA [43,44]. In conclusion, most of the metabolites we found to associate with disease inflammation have been described before, either in vivo or in vitro, and in most cases have been shown to be related to inflammation in RA in this study.

Although DAS28 has been extensively validated and is the most widely used instrument in clinical trials as well as in daily practice, it should also be noted that it has been claimed to be a rather instable monitoring instrument in RA patients with stable disease [45,46]. Two recently developed and validated instruments–the simplified disease activity index (SDAI) [14] and the clinical disease activity index (CDAI) [47], have been suggested to be used instead in clinical practice. Especially SDAI recently gained more relevance due to its inclusion in the American College of Rheumatism/EULAR remission criteria [48]. It has been reported that DAS28, SDAI, and CDAI do not result in the same classification of patients [49]. These newer instruments may reflect disease activity to a better extent, however SDAI and CDAI could not be used in our analyses because the Evaluator Global Assessment, a component included in both SDAI and CDAI, was not systemically collected in the BiOCURA study.

In this study, only metabolites at baseline were used to predict response and cross-sectionally investigate biomarkers for disease activity. As for the latter aim, investigating the change over time of metabolites might gain more insight in the most relevant metabolites regarding disease activity than any cross-sectional study, as they reflect prospective changes in RA patients.

## Conclusions

Metabolomic profiling is a powerful technique, which can be applied to analyze a wide range of metabolites from small sample volumes. Therefore, it has the potential of identification of biomarkers and increasing the understanding of the metabolic pathogenic pathways involved in a disease, such as RA. In the present study, we employed metabolic profiling to identify candidate metabolites to predict clinical response and assessed associations between metabolites and disease activity of RA. Because subjects were selected from an observational study, the heterogeneity needed to be adjusted for possible influential factors in all analyses. Yet, regardless of the possible absence of important influential factors, we showed that the predictive ability of a model can be quite high (AUC > 0.8) in a heterogeneous setting like clinical practice. As for its potential use, metabolites allow a better identification of non-responders on top of clinical parameters, which is of added value in determining the most fitting treatment for each individual patient. Further external validation is needed to assess the robustness of these findings and its potential value for clinical application.

This is the first time that serum metabolomic profiles analyzed by LC-MS have been demonstrated to predict therapeutic response to biological treatment in RA. It would be worth studying how these metabolites could be used for predicting patients’ response with external validation in a more homogenous cohort and thereby potentially optimize the treatment strategy for patients with RA.

## Supporting Information

S1 Fig Scatter plot of the clinical model between good responders and non-responders to TNFi therapy. Patients were groups according to their observed responses on x-axis; y-axis represents the predicted probability calculated by the regression. The pseudo R-square, as a measure for the explained variance in the observed response by the model was 0.147 (COX & Snell).
(TIF)

S2 Fig Scatter plot of the combined model between good responders and non-responders to TNFi therapy. Patients were groups according to their observed responses on x-axis; y-axis represents the predicted probability calculated by the regression. The pseudo R-square, as a measure for the explained variance in the observed response by the model was 0.433 (COX & Snell).
(TIF)

S3 Fig ROC curve for the clincial model containing non-, moderate- and good responders to TNFi therapy. The AUC-ROC was 0.641 (95% CI: 0.548–0.734).
(TIF)

S4 Fig ROC curve for the combined model non-, moderate- and good responders to TNFi therapy. The AUC-ROC was 0.760 (95% CI: 0.682–0.837).
(TIF)

S1 Table Baseline characteristics of all selected subjects (n = 231), and split for all EULAR good-responders and non-responders (n = 80 each). (PDF)

S2 Table Previously and currently used treatments of all selected subjects and split for responders and non-responders. (PDF)

S3 Table List of relative standard deviations (RSD) for all 139 measured metabolites. (PDF)

S4 Table List of detected metabolites in lipids analysis. (PDF)

S5 Table List of detected metabolites in oxylipins analysis. (PDF)

S6 Table List of detected metabolites in amines analysis. (PDF)

S7 Table Classification table of predicted good- and non-responders and observed good- and non-responders. (PDF)

S8 Table Net reclassification index of prediction models for sensitivity analysis. (PDF)

S9 Table Metabolites cross-sectionally associated with either baseline DAS28, ESR or CRP (*p* < 0.05) based on the complete cohort of bDMARD users (n = 231). (PDF)
