# Supplementary figures and images for: Exploring the Inflammatory Metabolomic Profile to Predict Response to TNF-α Inhibitors in Rheumatoid Arthritis
Source: PLoS One. 2016 Sep 15;11(9):e0163087. doi: 10.1371/journal.pone.0163087 (PMC5025050; doi:10.1371/journal.pone.0163087)

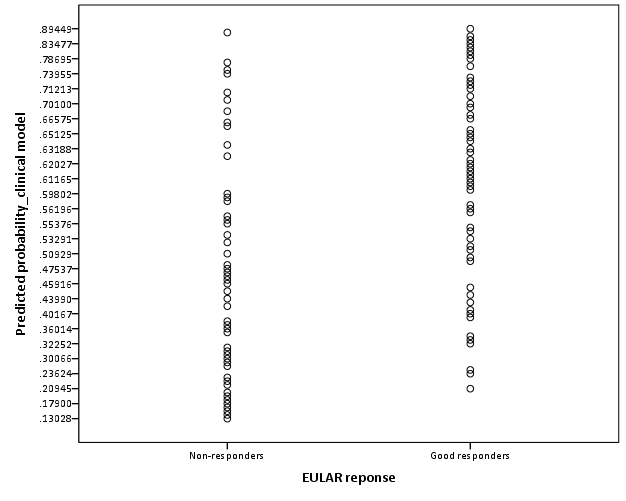

Supplement: S1 Fig — Patients were groups according to their observed responses on x-axis; y-axis represents the predicted probability calculated by the regression. The pseudo R-square, as a measure for the explained variance in the observed response by the model was 0.147 (COX & Snell). (TIF) [file pone.0163087.s001.tif]

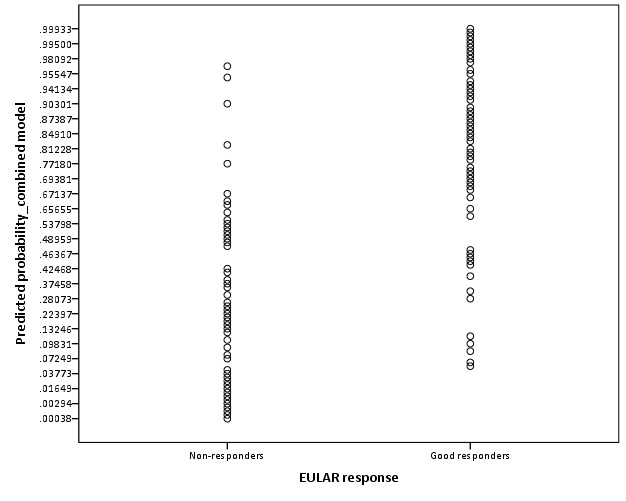

Supplement: S2 Fig — Patients were groups according to their observed responses on x-axis; y-axis represents the predicted probability calculated by the regression. The pseudo R-square, as a measure for the explained variance in the observed response by the model was 0.433 (COX & Snell). (TIF) [file pone.0163087.s002.tif]

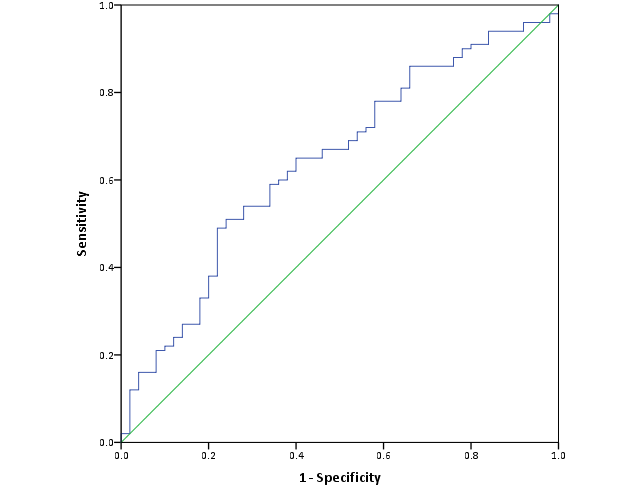

Supplement: S3 Fig — The AUC-ROC was 0.641 (95% CI: 0.548–0.734). (TIF) [file pone.0163087.s003.tif]

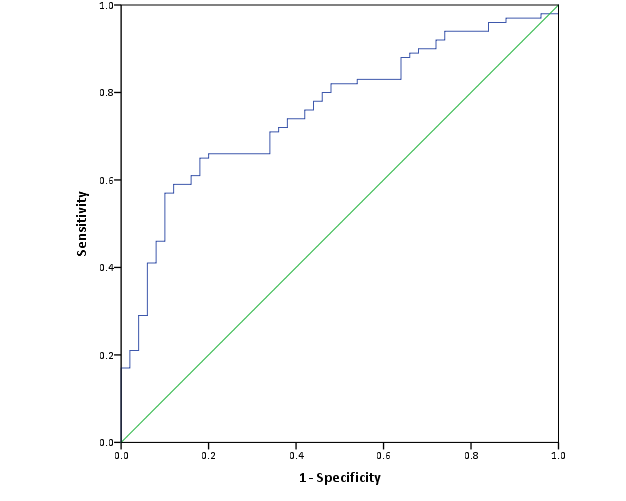

Supplement: S4 Fig — The AUC-ROC was 0.760 (95% CI: 0.682–0.837). (TIF) [file pone.0163087.s004.tif]
