# Supplementary material for: Exploring the Inflammatory Metabolomic Profile to Predict Response to TNF-α Inhibitors in Rheumatoid Arthritis
Source: PLoS One. 2016 Sep 15;11(9):e0163087. doi: 10.1371/journal.pone.0163087 (PMC5025050; doi:10.1371/journal.pone.0163087)
Supplement: S1 Table — (PDF) [file pone.0163087.s005.pdf]

Table S1. Baseline characteristics of all selected subjects (n=231), and split for all EULAR good-responders and non-responders (n=80 each).

	All (n=231)	Non-responders (n=80)	Good responders (n=80)	p-value
**Gender, female, n (%)**	172 (74.5)	64 (80.0)	57 (71.3)	0.28
**Menopausal status of females, n(%)**				0.82
**Pre-menopause**	55 (23.8)	22 (34.4)	22 (38.6)	
**Post-menopause**	107 (46.3)	36 (56.3)	33 (57.9)	
**Unknown**	10 (4.3)	6 (9.4)	2 (3.5)	
**Age, years, mean (SD)**	54.5 (13.1)	52.8 (13.5)	52.8 (13.0)	1.00
**Disease duration, years, mean (SD)**	7.0 (3.0-14.0)	6.0 (3.0-12.0)	7.0 (3.0-12.8)	0.79
**Smoking, currently**	53 (22.9)	18 (22.5)	18 (22.5)	1.00
**Alcohol, >7 units/week**	39 (17.0)	9 (11.3)	16 (20.3)	0.13
**BMI, kg/m^2, mean (SD)**	26.6 (5.1)	27.1 (5.1)	26.5 (4.9)	0.47
**Positive RF, n(%)**	151 (65.4)	47 (58.8)	59 (73.8)	0.07
**Positive ACPA, n(%)**	161 (69.7)	50 (62.5)	59 (73.8)	0.17
**CRP, mg/dL, median (IQR)**	6.0 (3.0-16.5)	5.0 (2.0-10.5)	8.0 (3.0-20.3)	0.05
**Baseline DAS28, mean (SD)**	4.7 (1.1)	4.4 (1.2)	4.6 (0.9)	0.18
TJC, median (IQR)	7.0 (2.0-14.0)	7.0 (2.0-14.0)	7.0 (3.0-12.8)	0.71
SJC, median (IQR)	2.0 (0.0-4.0)	1.0 (0.0-3.0)	2.0 (0.0-4.0)	0.06
ESR, mm/h, median IQR	21.0 (21.0-39.0)	18.0 (6.0-34.0)	18.0 (10.0-36.5)	0.53
VAS-GH, mean (SD)	58.2 (22.9)	58.2 (23.1)	55.8 (22.6)	0.50

Descriptive statistics are expressed as number (%) for dichotomized variables, and mean ± standard deviation (SD) and median and interquartile range (IQR) for respectively normally and non-normally distributed variables. The p-value is calculated for the difference between responders and non-responders. ACPA, anti-citrullinated protein antibody; BMI, body mass index; DAS28, disease activity score based on 28 joint count; ESR, erythrocyte sedimentation rate; IQR: interquartile range; RF: rheumatoid factor; SJC, 28 swollen joint count; TJC, 28 tender joint count; VAS-GH, 100mm visual analogue scale on general health.
