# Supplementary material for: Exploring the Inflammatory Metabolomic Profile to Predict Response to TNF-α Inhibitors in Rheumatoid Arthritis
Source: PLoS One. 2016 Sep 15;11(9):e0163087. doi: 10.1371/journal.pone.0163087 (PMC5025050; doi:10.1371/journal.pone.0163087)
Supplement: S2 Table — (PDF) [file pone.0163087.s006.pdf]

Table S2. Previously and currently used treatments of all selected subjects (n = 231) and split for good responders and non-responders (n = 80 each). P-values were calculated based on chi-square/fisher exact.

Treatment		All (n=231)	Non-responder (n=80)	Good responders (n=80)	*p*-value
**No. of previously used bDMARDs, n (%)**					0.16
	0 (naïve)	116 (50.2)	37 (46.3)	49 (61.3)	
	1	56 (24.2)	24 (30.0)	17 (21.3)	
	>1	59 (25.5)	17 (23.8)	14 (17.5)	
**Initiated treatment, n (%)**					0.10
	Adalimumab	70 (30.3)	25 (31.3)	27 (33.8)	
	Etanercept	60 (26.0)	22 (27.5)	23 (28.8)	
	Golimumab	23 (10.0)	7 (8.8)	5 (6.3)	
	Infliximab	10 (4.3)	6 (7.5)	3 (3.8)	
	Certolizumab	10 (4.3)	4 (5.0)	2 (2.5)	
	Abatacept	19 (8.2)	8 (10.0)	3 (3.8)	
	Tocilizumab	25 (10.8)	3 (3.8)	14 (17.5)	
	Rituximab	14 (6.1)	5 (6.3)	3 (3.8)	
**Concomitant DMARDs, n (%)**		217 (93.9)	76 (95.0)	75 (93.8)	1.00
	Methotrexate	166 (71.9)	55 (68.8)	64 (80)	0.15
	Hydroxychloroquine	50 (21.6)	18 (22.5)	19 (23.8)	1.00
	Leflunomide	28 (12.1)	11 (13.8)	5 (6.3)	0.19
	Glucocorticoids	93 (40.3)	36 (45.0)	26 (32.5)	0.14
**Concomitant non-DMARDs, n (%)**					
	Statins	34 (14.7)	14 (17.5)	6 (7.5)	0.09
	Anti-osteoporosis	101 (43.7)	37 (46.3)	32 (40.0)	0.52
	Anti-hypertention	68 (29.4)	25 (31.2)	17 (21.3)	0.21
	NSAIDs	142 (61.5)	45 (56.3)	49 (61.3)	0.63

P-values were calculated based on chi-square/fisher exact.

DMARDs: biological DMARDs, DMARDs: disease-modifying antirheumatic drugs.
