# Supplementary material for: Exploring the Inflammatory Metabolomic Profile to Predict Response to TNF-α Inhibitors in Rheumatoid Arthritis
Source: PLoS One. 2016 Sep 15;11(9):e0163087. doi: 10.1371/journal.pone.0163087 (PMC5025050; doi:10.1371/journal.pone.0163087)
Supplement: S3 Table — (PDF) [file pone.0163087.s007.pdf]

Table S3. List of relative standard deviations (RSD) for all 139 measured metabolites

Phospholipids*	RSD	Fatty acids*	RSD	Oxylipins	RSD	Amines	RSD
LPE (16:0)	5%	FA (14:0)	8%	9,10-DiHOME	6%	Ethanolamine	6%
LPE (18:0)	3%	FA (14:1)	26%	12,13-DiHOME	6%	Ornithine	3%
LPE (18:1)	5%	FA (15:0)	23%	TXB2	7%	Lysine	5%
LPE (18:2)	5%	FA (16:0)	7%	13-HODE	7%	Glycine	4%
LPE (20:3- ω3ω6)	8%	FA (16:1)	5%	14-HDoHE	8%	Sarcosine	10%
LPE (20:4)	5%	FA (17:0)	12%	13-HDoHE	8%	Alanine	3%
LPE (20:5)	10%	FA (17:1)	11%	14,15-DiHETrE	10%	3-Aminoisobutyric acid	6%
LPE (22:5-ω3)	9%	FA(18:0)	9%	13,14-dihydro-PGF2a	11%	α-aminobutyric acid	3%
LPE (22:5-ω6)	22%	FA (18:1)	3%	9-HODE	11%	Serine	4%
LPE (22:6)	4%	FA (18:2)	3%	19,20-DiHDPA	12%	Cystathionine	7%
sn2-LPC (14:0) [a]	11%	FA (18:3-ω3ω6)	3%	11-HETE	13%	Proline	14%
sn1-LPC (14:0) [b]	6%	FA (20:0)	18%	15S-HETrE	14%	Valine	4%
sn1-LPC (15:0)	7%	FA (20:1)	8%	10-HDoHE	15%	Threonine	4%
sn2-LPC (16:0)	7%	FA (20:2)	13%	5,6-DiHETrE	16%	Cysteine	6%
sn1-LPC (16:0)	2%	FA (20:3-ω3ω6)	6%	11,12-DiHETrE	16%	Taurine	4%
sn2-LPC (16:1)	8%	FA (20:3-ω9)	20%	LTB4	17%	Pipecolic acid	6%
sn1-LPC (16:1)	6%	FA (20:4-ω6)	4%	8,9-DiHETrE	17%	Isoleucine	3%
sn2-LPC (18:0)	3%	FA (20:5-ω3)	6%	9,10-EpOME	17%	Leucine	4%
sn1-LPC (18:0)	2%	FA (22:4)	6%	5-HETE	18%	4-Hydroxyproline	3%
sn2-LPC (18:1)	4%	FA (22:5-ω3)	3%	PGE2	18%	Asparagine	3%
sn1-LPC (18:1)	6%	FA (22:5-ω6)	4%	17,18-DiHETE	18%	Glycylglycine	4%
sn2-LPC (18:2)	8%	FA (22:6)	6%	12-HETE	18%	Aspartic acid	4%
sn1-LPC (18:2)	7%	FA (24:0)	15%	20-carboxy-LTB4	18%	s-Methylcysteine	5%
sn2-LPC (18:3-ω3ω6)	20%	FA (24:1)	9%	TXB1	19%	Homocysteine	7%
sn1-LPC (18:3-ω3ω6)	7%			12,13-EpOME	19%	O-Phosphoethanolamine	14%
sn1-LPC (19:0)	24%			9-HOTrE	20%	Glutamine	4%
sn1-LPC (20:1)	13%			11-HDoHE	20%	Glutamic acid	4%
sn1-LPC (20:2)	7%			9,12,13-TriHOME	20%	Methionine	4%
sn2-LPC (20:3-ω3ω6)	9%			TXB3	21%	Histidine	5%
sn1-LPC (20:3-ω3ω6)	9%			8-HETE	22%	Methionine sulfoxide	9%
sn1-LPC (20:3-ω9)	17%			12S-HHTrE	30%	Phenylalanine	3%
sn2-LPC (20:4)	6%					1-Methylhistidine	15%
sn1-LPC (20:4)	8%					Arginine	8%
sn2-LPC (20:5)	9%					Citrulline	3%
sn1-LPC (20:5)	8%					Serotonin	11%
sn1-LPC (22:4)	9%					Tyrosine	4%
sn1-LPC (22:5-ω3) [d]	8%					Tryptophan	4%
sn1-LPC (22:5-ω6)	9%					Kynurenine	4%

sn2-LPC (22:6)	8%						γ-glutamylalanine	12%
sn1-LPC (22:6)	7%						Glutathione	10%
LPC (O-16:0) [c]	6%							
LPC (O-18:0)	12%							
LPC (O-18:1)	6%							
LPC (O-18:2)	19%							

The RSDs were calculated on the metabolites in the QC samples. The "sn1-" or "sn2-" prefix is used to indicate the position of the fatty acid chain esterified to the glycerol backbone of a lysophospholipid. The 'O-' prefix is used to identify a plasmalogen lysophospholipid, where the fatty acid chain is attached via a vinyl ether linkage to the glycerol backbone. The 'ω3', 'ω6', 'ω9' are used to indicate double bond (C=C) at the third, sixed or ninth carbon atom from the end of the fatty acid chain.

*The number in brackets represent the long-chain fatty acid attached in the lysophospholipids (e.g. FA(14:0) represents a fatty acid with 14 carbons and 0 double bonds).

LPE: lysophosphatidylethanolamine; LPC: lysophospholipids; FA: fatty acid; LTB4: leukotriene B_4; DiHDPA: dihydroxy-docosapentaenoic acid; DiHETE: dihydroxy-eicosatetraenoic acid; DiHETrE: dihydroxy-eicosatrienoic acid; DiHOME: dihydroxy-octadecenoic acid; PGF2a: prostaglandin F2α; EpOME: epoxy-octadecenoic acid; HDoHE: hydroxyl-docosahexaenoic acid; HEPE: hydroxy-eicosapentaenoic acid; HETE: hydroxyicosatetraenoic acid; HETrE: hydroxy-eicosatrienoic acid; HHTrE: hydroxy-heptadecatrienoic acid; HODE: hydroxy-octadecadienoic acid; HOTrE: hydroxy-octadecatrienoic acid; KODE: oxo-octadecadienoic acid; TriHOME: trihydroxy-octadecenoic acid.
