# Supplementary material for: Exploring the Inflammatory Metabolomic Profile to Predict Response to TNF-α Inhibitors in Rheumatoid Arthritis
Source: PLoS One. 2016 Sep 15;11(9):e0163087. doi: 10.1371/journal.pone.0163087 (PMC5025050; doi:10.1371/journal.pone.0163087)
Supplement: S4 Table — (PDF) [file pone.0163087.s008.pdf]

Table S4 List of detected metabolites in lipids analysis

Lipid Class	Lipid Maps	Metabolite species	Amount (n)
Free fatty acids (FA)	FA01	14:0, 14:1, 15:0, 16:0, 16:1, 17:0, 17:1,18:0, 18:1, 18:2, 18:3-ω3ω6, 20:0, 20:1, 20:2, 20:3-ω3ω6, 20:3-ω9, 20:4-ω6, 20:5-ω3, 22:4, 22:5-ω3, 22:5-ω6, 22:6, 24:0, 24:1	24
Lysophosphatidylcholine (LPC)	GP0105	*sn1:* , 14:0, 15:0, 16:0, 16:1, 18:0, 18:1, 18:2, 18:3-ω3ω6, 19:0, 20:1, 20:2, 20:3-ω3ω6, 20:3-ω9, 20:4, 20:5, 22:4, 22:5-ω3, 22:5-ω6, 22:6<br>*sn2:* , 14:0, 16:0, 16:1, 18:0, 18:1, 18:2, 18:3-ω3ω6, 20:3-ω3ω6, 20:4, 20:5, 22:6	30
Lysophosphatidylethanolamine (LPE)	GP0205	16:0, 18:0, 18:1, 18:2, 20:3- ω3ω6, 20:4, 20:5, 22:5-ω3, 22:5-ω6, 22:6	10
Plasmalogen Lysophosphatidylcholine (pLPC)	GP0106	C16:0; C18:0; C18:1; C18:2	4
