# Supplementary material for: Exploring the Inflammatory Metabolomic Profile to Predict Response to TNF-α Inhibitors in Rheumatoid Arthritis
Source: PLoS One. 2016 Sep 15;11(9):e0163087. doi: 10.1371/journal.pone.0163087 (PMC5025050; doi:10.1371/journal.pone.0163087)
Supplement: S5 Table — (PDF) [file pone.0163087.s009.pdf]

Table S5 List of detected metabolites in oxylipins analysis.

Metabolite	Systematic name	Formula	Lipid Maps ID
10-HDoHE	(+/-)-10-hydroxy-4Z,7Z,11E,13Z,16Z,19Z-docosahexaenoic acid	C22H32O3	LMFA04000027
11,12-DiHETrE	(±)11,12-dihydroxy-5Z,8Z,14Z-eicosatrienoic acid	C20H34O4	LMFA03050008
11-HDoHE	(+/-)-11-hydroxy-4Z,7Z,9E,13Z,16Z,19Z-docosahexaenoic acid	C22H32O3	LMFA04000028
11-HETE	11R-hydroxy-5Z,8Z,12E,14Z-eicosatetraenoic acid	C20H32O3	LMFA03060028
12,13-DiHOME	(+/-)-12,13-dihydroxy-9Z,15Z-octadecadienoic acid	C18H32O4	LMFA02000046
12,13-EpOME	(+/-)-12(13)-epoxy-9Z-octadecenoic acid	C18H32O3	LMFA02000038
12-HETE	12-hydroxy-5Z,8Z,10E,14Z-eicosatetraenoic acid	C20H32O3	LMFA03060088
12S-HHTrE	12S-hydroxy-5Z,8E,10E-heptadecatrienoic acid	C17H28O3	LMFA03050002
13,14-dihydro-PGF2a	9S,11R,15S-trihydroxy-5Z-prostenoic acid	C20H36O5	LMFA03010079
13-HDoHE	(+/-)-13-hydroxy-4Z,7Z,10Z,14E,16Z,19Z-docosahexaenoic acid	C22H32O3	LMFA04000029
13-HODE	13S-hydroxy-9Z,11E-octadecadienoic acid	C18H32O3	LMFA01050349
14,15-DiHETrE	14,15-dihydroxy-5Z,8Z,11Z-eicosatrienoic acid	C20H34O4	LMFA03050010
14-HDoHE	(+/-)-14-hydroxy-4Z,7Z,10Z,12E,16Z,19Z-docosahexaenoic acid	C22H32O3	LMFA04000030
15S-HETrE	15S-hydroxy-8Z,11Z,13E-eicosatrienoic acid	C20H34O3	LMFA03050007
17,18-DiHETE	(+/-)-17,18-dihydroxy-5Z,8Z,11Z,14Z-eicosatetraenoic acid	C20H32O4	LMFA03060078
19,20-DiHDPA	(±)19,20-dihydroxy-4Z,7Z,10Z,13Z,16Z-docosapentaenoic acid	C22H34O4	LMFA04000043
20-carboxy-LTB4	5S,12R-dihydroxy-6Z,8E,10E,14Z-eicosatetraene-1,20-dioic acid	C20H30O6	LMFA03020016
5,6-DiHETrE	5S,6S-dihydroxy-7E,9E,11Z,14Z-eicosatetraenoic acid	C20H32O4	LMFA03060018
5-HETE	5S-hydroxy-6E,8Z,11Z,14Z-eicosatetraenoic acid	C20H32O3	LMFA03060002
8,9-DiHETrE	8,9-dihydroxy-5Z,11Z,14Z-eicosatrienoic acid	C20H34O4	LMFA03050006
8-HETE	(±)8-hydroxy-5Z,9E,11Z,14Z-eicosatetraenoic acid	C20H32O3	LMFA03060086
9,10-DiHOME	9,10-dihydroxy-12Z-octadecenoic acid	C18H34O4	LMFA01050350
9,10-EpOME	(+/-)-9(10)-epoxy-12Z-octadecenoic acid	C18H32O3	LMFA02000037
9,12,13-TriHOME	9S,12S,13S-trihydroxy-10E-octadecenoic acid	C18H34O5	LMFA02000014
9-HODE	9S-hydroxy-10E,12Z-octadecadienoic acid	C18H32O3	LMFA01050278
9-HOTrE	9S-hydroxy-10E,12Z,15Z-octadecatrienoic acid	C18H30O3	LMFA02000024
LTB4	5S,12R-dihydroxy-6Z,8E,10E,14Z-eicosatetraenoic acid	C20H32O4	LMFA03020001
PGE2	9-oxo-11R,15S-dihydroxy-5Z,13E-prostadienoic acid; Prostin E2	C20H32O5	LMFA03010003
TXB1	9S,11,15S-trihydroxy-thrombox-13E-enoic acid	C20H36O6	LMFA03030008
TXB2	9S,11,15S-trihydroxy-thromboxa-5Z,13E-dien-1-oic acid	C20H34O6	LMFA03030002
TXB3	9S,11,15S-trihydroxy-thromboxa-5Z,13E, 17Z-trien-1-oic acid	C20H32O6	LMFA03030006
