# Supplementary material for: Exploring the Inflammatory Metabolomic Profile to Predict Response to TNF-α Inhibitors in Rheumatoid Arthritis
Source: PLoS One. 2016 Sep 15;11(9):e0163087. doi: 10.1371/journal.pone.0163087 (PMC5025050; doi:10.1371/journal.pone.0163087)
Supplement: S6 Table — (PDF) [file pone.0163087.s010.pdf]

Table S6 List of detected metabolites in amines analysis

Metabolite	Chemical formula	HMDB ID	InChI Key
1-Methylhistidine	$C_7H_{11}N_3O_2$	HMDB00001	BRMWTNUJHUMWMS-LURJTMIESA-N
3-Aminoisobutyric acid	$C_4H_9NO_2$	HMDB00452	QWCKQJZIFLGMSD-VKHMYHEASA-N
4-Hydroxyproline	$C_5H_9NO_3$	HMDB00725	PMMYEEVYMWASQN-DMTCNVIQSA-N
Alanine	$C_3H_7NO_2$	HMDB00161	QNAYBMKLOCPYGJ-REOHCLBHSA-N
α-aminobutyric acid	$C_4H_9NO_2$	HMDB03911	QCHPKSFMDHP*SN*R-UHFFFAOYSA-N
Arginine	$C_6H_{14}N_4O_2$	HMDB00517	ODKSFYDXXFIFQN-BYPYZUCNSA-N
Asparagine	$C_4H_8N_2O_3$	HMDB00168	DCXYFEDJOCDNAF-REOHCLBHSA-N
Aspartic acid	$C_4H_7NO_4$	HMDB00191	CKLJMWTZIZZHCS-REOHCLBHSA-N
Citrulline	$C_6H_{13}N_3O_3$	HMDB00904	RHGKLRLOHDJJDR-BYPYZUCNSA-N
Cystathionine	$C_7H_{14}N_2O_4S$	HMDB00099	ILRYLPWNYFXEMH-WHFBIAKZSA-N
Cysteine	$C_3H_7NO_2S$	HMDB00574	XUJNEKJLAYXESH-REOHCLBHSA-N
Ethanolamine	C_2H_7NO	HMDB00149	HZAXFHJVJLSVMW-UHFFFAOYSA-N
Glutamic acid	$C_5H_9NO_4$	HMDB00148	WHUUTDBJXJRKMK-VKHMYHEASA-N
Glutamine	$C_5H_{10}N_2O_3$	HMDB00641	ZDXPYRJPNDTMRX-VKHMYHEASA-N
Glutathione	$C_{10}H_{17}N_3O_6S$	HMDB00125	RWSXRVCMGQZWBV-WDSKDSINSA-N
Glycine	$C_2H_5NO_2$	HMDB00123	DHMQDGOQFOQNFH-UHFFFAOYSA-N
Glycylglycine	$C_4H_8N_2O_3$	HMDB11733	YMAWOPBAYDPSLA-UHFFFAOYSA-N
Histidine	$C_5H_9N_3$	HMDB00870	NTYJJOPFIAHURM-UHFFFAOYSA-N
Homocysteine	$C_4H_9NO_2S$	HMDB00742	FFFHZYDWPBMWHY-UHFFFAOYSA-N
Isoleucine	$C_6H_{13}NO_2$	HMDB00172	AGPKZVBTJJNPAG-WHFBIAKZSA-N
Kynurenine	$C_{10}H_{12}N_2O_3$	HMDB00684	YGPSJZOEDVAXAB-QMMMGPOBSA-N
Leucine	$C_6H_{13}NO_2$	HMDB00687	ROHFNLRQFUQHCH-YFKPBYRVSA-N
Lysine	$C_6H_{14}N_2O_2$	HMDB00182	KDXKERNSBIXSRK-YFKPBYRVSA-N
Methionine	$C_5H_{11}NO_2S$	HMDB00696	FFEARJCKVFRZRR-BYPYZUCNSA-N
Methionine sulfoxide	$C_5H_{11}NO_3S$	HMDB02005	QEFRNWWLZKMPFJ-UHFFFAOYSA-N
O-Phosphoethanolamine	$C_2H_8NO_4P$	HMDB00224	SUHOOTKUPISOBE-UHFFFAOYSA-N
Ornithine	$C_5H_{12}N_2O_2$	HMDB00214	AHLPHDHHMVZTML-BYPYZUCNSA-N
Phenylalanine	$C_9H_{11}NO_2$	HMDB00159	COLNVLDHVKWLRT-QMMMGPOBSA-N
Pipecolic acid	$C_6H_{11}NO_2$	HMDB00716	HXEACLLIILLPRG-YFKPBYRVSA-N
Proline	$C_5H_9NO_2$	HMDB00162	ONIBWKKTOPOVIA-BYPYZUCNSA-N
Sarcosine	$C_3H_7NO_2$	HMDB00271	FSYKKLYZXJ*SN*PZ-UHFFFAOYSA-N
Serine	$C_3H_7NO_3$	HMDB00187	MTCFGRXMJLQNBG-REOHCLBHSA-N
Serotonin	$C_{10}H_{12}N_2O$	HMDB00259	QZAYGJVTTNCVMB-UHFFFAOYSA-N
s-Methylcysteine	$C_4H_9NO_2S$	HMDB02108	IDIDJDIHTAOVLG-VKHMYHEASA-N
Taurine	$C_2H_7NO_3S$	HMDB00251	XOAAWQZATWQOTB-UHFFFAOYSA-N
Threonine	$C_4H_9NO_3$	HMDB00167	AYFVYJQAPQTCCC-GBXIJSLDSA-N
Tryptophan	$C_{11}H_{12}N_2O_2$	HMDB00929	QIVBCDIJIAJPQS-VIFPVBQESA-N
Tyrosine	$C_9H_{11}NO_3$	HMDB00158	OUYCCCASQSFEME-QMMMGPOBSA-N

Valine	C5H11NO2	HMDB00883	KZ*SN*JWFQEVHDMF-BYPYZUCNSA-N
γ-glutamylalanine	C10H17N3O6	HMDB11738	JBFYFLXEJFQWMU-WDSKDSINSA-N
