# Supplementary material for: Exploring the Inflammatory Metabolomic Profile to Predict Response to TNF-α Inhibitors in Rheumatoid Arthritis
Source: PLoS One. 2016 Sep 15;11(9):e0163087. doi: 10.1371/journal.pone.0163087 (PMC5025050; doi:10.1371/journal.pone.0163087)
Supplement: S7 Table — (PDF) [file pone.0163087.s011.pdf]

Table S7. Classification table of predicted good- and non-responders and observed good- and non-responders.

	Predicted \ Observed	Good responder	Non-responders	Sensitivity	Specificity	PPV	NPV	MR
Clinical model	Good response	43	20	0.782	0.600	0.683	0.714	0.305
	Non-response	12	30					
Combined model	Good response	39	5	0.709	0.900	0.887	0.738	0.200
	Non-response	16	45					

PPV: positive predictive values; NPV: negative predictive values; MR: misclassification rate.
