# Supplementary material for: Exploring the Inflammatory Metabolomic Profile to Predict Response to TNF-α Inhibitors in Rheumatoid Arthritis
Source: PLoS One. 2016 Sep 15;11(9):e0163087. doi: 10.1371/journal.pone.0163087 (PMC5025050; doi:10.1371/journal.pone.0163087)
Supplement: S8 Table — (PDF) [file pone.0163087.s012.pdf]

Table S8. Net reclassification index of prediction models for sensitivity analysis.

Observed response	Predicted by clinical model	Predicted by combined model	
		**Non-response**	**Response**
Non-responders (n=50)	**Non-response**	30 (equal)	0 (worsening)
	**Response**	15 (improvement)	5 (equal)
Responders (n=100)	**Non-response**	23 (equal)	12 (improvement)
	**Response**	20 (worsening)	45 (equal)
