# Supplementary material for: Exploring the Inflammatory Metabolomic Profile to Predict Response to TNF-α Inhibitors in Rheumatoid Arthritis
Source: PLoS One. 2016 Sep 15;11(9):e0163087. doi: 10.1371/journal.pone.0163087 (PMC5025050; doi:10.1371/journal.pone.0163087)
Supplement: S9 Table — (PDF) [file pone.0163087.s013.pdf]

Table S9. Metabolites cross-sectionally associated with either baseline DAS28, ESR or CRP ($p < 0.05$) based on the complete cohort of bDMARD users (n=231).

	**LnCRP**		**DAS28**		**lnESR**	
	Coefficients	*p*-value	Coefficients	*p*-value	Coefficients	*p*-value
sn2-LPC (14:0)	-.666	.004	-.066	.323	-.604	.002
sn1-LPC (14:0)	-.713	.000	-.080	.255	-.606	.000
sn2-LPC (16:1)	-.525	.194	-.122	.098	-.759	.000
sn1-LPC (18:3-w3w6)	-.223	.487	.048	.564	-.646	.000
sn1-LPC (20:5)	-1.023	.045	-.007	.944	-.556	.138
sn1-LPC (16:1)	-.633	.099	-.086	.213	-.744	.000
sn2-LPC (18:2)	-.396	.078	-.785	.001	-.468	.022
sn1-LPC (15:0)	-.960	.002	-1.176	0.243	-.578	.002
sn1-LPC (18:2)	-.397	.134	-.753	.001	-.437	.031
sn1-LPC (20:4)	-.418	.049	-.056	.475	-.357	.029
sn2-LPC (20:3)-w3w6	-.093	.510	-.071	.280	-.442	.028
LPE (22:5-w3)	-.561	.038	-.071	.450	-.300	.058
sn1-LPC (22:5-w3)	-.710	.009	-.109	.167	-.573	.003
sn1-LPC (20:3-w3w6)	-.268	.081	-.090	.200	-.513	.006
LPE (22:5-w6)	-.437	.223	-.730	.012	-.602	.002
sn1-LPC (22:5-w6)	-.379	.004	-.725	.000	-.596	.000
sn1-LPC (18:1)	-.355	.182	-.081	.314	-.439	.032
sn1-LPC (20:3-w9)	.000	.999	-.132	.059	-.482	.004
sn1-LPC (22:4)	-.488	.005	-.049	.517	-.677	.000
LPC (O-16:0)	-.289	.015	-.729	.000	-.289	.059
LPC (O-18:2)	-.441	.326	-.103	.155	-.778	.005
sn1-LPC (20:2)	-.351	.077	-.011	.875	-.506	.010
sn1-LPC (19:0)	-.297	.517	-.662	.004	-.276	.147
LPC (O-18:0)	-.537	.006	-.057	.520	-.212	.177
FA (14:1)	.627	.000	.607	.000	.444	.000
FA (14:0)	.278	.037	.059	.424	.407	.000
FA (20:5-w3)	.025	.872	.009	.918	.496	.000
FA (18:3-w3w6)	.402	.007	.090	.263	.371	.001
FA (16:1)	.441	.006	.640	.000	.477	.000
FA (15:0)	.232	.034	.036	.633	.290	.003
FA (22:6)	.434	.169	-.012	.879	.726	.000
FA (20:4-w6)	.095	.574	.013	.846	.482	.000
FA (18:2)	.443	.039	.067	.314	.459	.000
FA (22:5-w3)	.363	.111	.590	.000	.489	.000
FA (17:1)	.475	.003	.052	.540	.450	.000

FA (22:5-w6)	.380	.198	-.008	.917	.571	.000
FA (20:3-w3w6)	.194	.206	-.012	.869	.502	.000
FA (16:0)	.244	.023	.421	.000	.331	.000
FA (20:3-w9)	.226	.013	.075	.814	-.045	.749
FA (18:1)	.547	.009	.583	.000	.471	.000
FA (22:4)	.438	.060	.597	.000	.460	.000
FA (17:0)	.220	.001	.353	.001	.215	.013
FA (20:2)	1.032	0.064	.002	.989	.406	.002
FA (18:0)	.175	.015	.351	.000	.223	.016
FA (20:1)	.451	.039	.093	.220	.427	.000
FA (24:1)	.349	.151	.529	.000	.369	.004
TXB3	.486	.000	.396	.183	.095	.436
TXB1	.404	.002	.937	.000	.596	.001
TXB2	.467	.002	.502	.213	.014	.931
9,12,13-TriHOME	-.081	.714	-.646	.073	-.505	.008
PGE2	.392	.015	.736	.021	.362	.017
13,14-dihydro-PGF2a	-.352	.008	-.769	.000	-.411	.060
19,20-DiHDPA	-.683	0.020	-.036	.924	.006	.984
14,15-DiHETrE	-.746	.000	.368	.190	.234	.457
11,12-DiHETrE	-.586	.006	.475	.051	.316	.205
9-HOTrE	.068	.686	.359	.020	.292	.019
8,9-DiHETrE	-.611	.005	-.216	.427	.070	.669
5,6-DiHETrE	-.997	.005	.255	.644	.054	.900
9-HODE	.012	.933	.255	.183	.260	.010
14-HDoHE	.427	.136	.749	.041	.548	.003
10-HDoHE	.238	.295	.675	.009	.645	.000
13-HDoHE	.270	.362	.648	.086	.580	.003
11-HETE	.396	.001	.895	.000	.420	.005
11-HDoHE	.242	.063	.860	.000	.526	.000
12-HETE	.463	.003	.708	.048	.412	.006
8-HETE	.243	.020	.654	.000	.413	.001
15S-HETrE	.281	.013	.730	.000	.392	.012
5-HETE	.273	.010	.729	.000	.498	.000
Ornithine	.483	.003	.061	.832	.024	.905
Sarcosine	-.386	.065	-.664	.003	-.558	.000
Alpha-aminobutyric acid	.181	.446	-.433	.035	-.166	.479
Proline	.284	.028	-.051	.573	.103	.373
Valine	.840	.000	-.017	.829	.268	.134
Threonine	.106	.678	-.205	.006	-.102	.655
Cysteine	.232	.107	.085	.392	.280	.017
Taurine	.626	.279	.124	.086	.461	.005

Isoleucine	.638	.001	.041	.606	.197	.218
Leucine	.642	.000	.323	.160	.190	.284
4-Hydroxyproline	.285	.024	-.007	.952	-.078	.425
Asparagine	-.033	.938	-.150	.024	-.146	.553
Glycylglycine	-.030	.944	-.164	.015	-.248	.324
Histidine	.051	.904	-.284	.000	-.113	.576
Arginine	.434	.012	-.059	.389	.211	.170
Serotonin	.977	.000	-.044	.655	.684	.000
Tryptophan	.791	.000	-.113	.216	-.108	.770
Kynurenine	.529	.026	.082	.338	-.004	.983
Gamma-glutamylalanine	.941	.000	-.111	.099	.209	.413
Glutathione	.388	.016	.433	.006	.472	.000
sn2-LPC (20:5)	-.728	.136	-.042	.671	-.435	.274
sn2-LPC (18:3-w3w6)	.138	.521	.147	.622	-.314	.073
LPE (20:5)	-.237	.409	.226	.459	.015	.947
sn2-LPC (20:4)	-.259	.127	-.064	.413	-.321	.058
sn2-LPC (22:6)	-.444	.085	-.081	.300	.088	.677
sn1-LPC (22:6)	-.456	.095	-.069	.369	.147	.561
LPE (18:2)	-.412	.338	-.026	.788	-.075	.770
LPE (22:6)	-.163	.429	.359	.135	.346	.070
LPE (20:4)	-.484	.177	-.008	.919	.070	.755
sn2-LPC (16:0)	-.124	.592	-.015	.852	-.118	.428
sn2-LPC (18:1)	-.110	.561	-.057	.451	-.298	.126
sn1-LPC (16:0)	-.191	.260	-.037	.626	-.180	.195
LPE (16:0)	-.283	.174	.021	.814	-.109	.401
LPE (20:3-w3w6)	-.359	.336	-.052	.550	-.023	.900
LPE (18:1)	-.316	.394	-.037	.747	.178	.439
sn2-LPC (17:0)	.133	.449	.203	.492	-.194	.199
sn2-LPC (18:0)	-.085	.550	-.048	.504	-.086	.464
LPC (O-18:1)	-.179	.174	-.022	.757	-.142	.198
LPE (18:0)	-.130	.396	.056	.547	.247	.125
sn1-LPC (18:0)	-.077	.542	-.051	.476	-.064	.556
sn1-LPC (20:1)	-.164	.356	.021	.713	-.036	.704
FA (20:0)	.301	.110	.035	.630	.191	.151
FA (24:0)	.184	.334	.076	.404	.144	.347
20-carboxy-LTB4	.102	.549	.262	.212	.130	.313
17,18-DiHETE	-.405	.053	.549	.081	.153	.624
LTB4	-.056	.691	.185	.550	.228	.188
12,13-DiHOME	.048	.823	.244	.205	.092	.479
9,10-DiHOME	-.108	.623	.133	.543	.089	.529
12S-HHTrE	.209	.592	.528	.189	-.172	.414

13-HODE	-.111	.446	.065	.774	.160	.141
12,13-EpOME	-.066	.665	.124	.476	.169	.065
9,10-EpOME	-.090	.554	.111	.536	.199	.059
Ethanolamine	.091	.878	.039	.620	.400	.102
Lysine	.191	.291	-.072	.246	.021	.915
Glycine	.009	.987	-.037	.673	-.230	.453
Alanine	.138	.602	-.097	.144	-.080	.722
3-Aminoisobutyric acid	-.052	.872	-.132	.095	-.308	.187
Serine	.084	.768	-.120	.152	-.265	.250
Cystathionine	-.178	.221	-.095	.355	-.233	.065
Pipecolic acid	.315	.070	-.183	.126	-.009	.963
Aspartic acid	.180	.562	.173	.585	.109	.577
s-Methylcysteine	.005	.988	.066	.528	.243	.327
Homocysteine	-.063	.839	.145	.072	.340	.498
O-Phosphoethanolamine	-.127	.493	.052	.588	.097	.430
Glutamine	.109	.751	-.049	.500	.228	.226
Glutamic acid	-.248	.413	.014	.861	-.304	.111
Methionine	.048	.844	-.052	.413	-.061	.735
Methionine sulfoxide	.651	.055	.040	.595	.283	.204
Phenylalanine	.242	.298	.052	.831	-.012	.951
1-Methylhistidine	.528	.113	.101	.123	.284	.127
Citrulline	-.309	.566	.285	.568	-.392	.326
Tyrosine	-.091	.763	-.014	.852	.157	.398

The log-transformed and autoscaled metabolites were put in a linear GEE on DAS28, ESR and CRP separately, together with possible influencing clinical parameters (age, gender, menopausal status, BMI, smoking status, alcohol consumption, concomitant csDMARDs (MTX, HCQ, LEF, GCs) and non-DMARDs (statins, antihypertensive drugs, bisphosphonates and NSAIDs). Shown are the regression coefficients and *p*-values per metabolite, only of those metabolites that associated to either DAS28, ESR or CRP.
